# Chemical and Microbiological Characterization of Freeze-Dried Superworm (*Zophobas morio* F.) Larvae Pretreated by Blanching and Ultrasound Treatment

**DOI:** 10.3390/molecules29225447

**Published:** 2024-11-19

**Authors:** Radosław Bogusz, Anna Onopiuk, Klara Żbik, Katarzyna Pobiega, Iga Piasecka, Małgorzata Nowacka

**Affiliations:** 1 Department of Food Engineering and Process Management, Institute of Food Sciences, Warsaw University of Life Sciences—SGGW, 159c Nowoursynowska Street, 02-776 Warsaw, Poland; 2 Department of Technique and Food Development, Institute of Human Nutrition Sciences, Warsaw University of Life Sciences—SGGW, 159c Nowoursynowska Street, 02-776 Warsaw, Poland; anna_onopiuk@sggw.edu.pl (A.O.);; 3 Department of Food Biotechnology and Microbiology, Institute of Food Sciences, Warsaw University of Life Sciences—SGGW, 159c Nowoursynowska Street, 02-776 Warsaw, Poland; katarzyna_pobiega@sggw.edu.pl; 4 Department of Chemistry, Institute of Food Sciences, Warsaw University of Life Sciences—SGGW, 159c Nowoursynowska Street, 02-776 Warsaw, Poland; iga_piasecka@sggw.edu.pl

**Keywords:** edible insects, drying, oil properties, mineral composition, allergen content, microbiological quality

## Abstract

Edible insects may solve the current problem of the greater demand for food for the world’s growing human population. This work aimed to examine the impact of blanching (BL) and ultrasound (US) at 20 and 50 °C as a pretreatment method on the chemical composition, mineral composition, FTIR spectra, presence of allergens and microorganisms, and properties of the isolated oil of freeze-dried superworm larvae. The US treatment resulted in significantly lower protein content (31.65–33.34 g/100 g d.m.) compared to untreated (36.38 g/100 g d.m.) and BL (37.72 g/100 g d.m.) samples. The study demonstrated that the US-treated insects exhibited a lower content of crustacean and mollusk allergens than the BL insects, and the lowest content of tested allergens was found in the US_50°C superworm larvae. Furthermore, oil isolated from US_50°C insects exhibited the lowest SFA and the highest PUFA content and the best prospective nutritional properties expressed through theoretical health indices. The presence of Enterobacteriaceae and anaerobic spore-forming bacteria was not detected in the tested insects, proving suitable microbiological quality. It appears that using US treatment is a promising alternative to traditional blanching of insects before drying.

## 1. Introduction

Drying is the most common technique used for food preservation and shelf-life extension. It facilitates transportation and long-term food storage, increases availability, and reduces food waste [1,2]. Nevertheless, drying causes undesirable changes in products’ color, texture, structure, and chemical composition, mainly using traditional methods based on hot air, such as convective drying [2,3]. A low-temperature method such as freeze-drying is frequently used due to the high overall attractiveness of dried products, e.g., high nutritional value, great rehydration ability, porous structure, attractive color and flavor [2,4,5], and limited chemical reactions occurring during storage due to the low water content in dried products, equal to 2–3% [4]. However, regardless of the method utilized, drying is a time-consuming and expensive operation. In this case, freeze-drying is obviously more costly than convective drying, given the equipment costs and the lowering and maintenance of temperature and pressure [5,6]. Diverse pre-treatment methods, both traditional and emerging non-thermal technologies, are being considered to optimize the drying process time and cost.

One of the promising non-thermal techniques with high potential to be implemented in the food industry is ultrasound treatment (US) based on low-frequency (20–100 kHz) and high-power ultrasonic waves. These waves may contract and expand on the material’s tissue particles, producing a huge amount of energy and interesting findings [7]. This effect, called the “sponge effect”, produces microchannels and changes the internal structure, e.g., providing an easier way to remove water from the material during drying. Ultrasound treatment may also cause cavitation, consisting of the explosion of gas bubbles due to sudden changes in pressure and temperature, causing irreversible cell damage, which may be used for microorganisms’ inactivation and facilitation of the cell content release into the environment [1,8,9]. Therefore, US pre-treatment can shorten the drying time, lower energy consumption and greenhouse gas emissions, and improve the dried product’s quality.

Edible insects may solve the current problem of greater demand for food for the world’s growing human population, which is expected to reach as many as 11 billion in 2100 [10]. Edible insects are a source of complete protein and all essential amino acids, as well as fat rich in unsaturated fatty acids, which are important in the human diet [11,12]. So far, four species of insects have been added to the list of novel foods in European Union countries. These are yellow mealworm (*Tenebrio molitor* L.), migratory locust (*Locusta migratoria* L.), house cricket (*Acheta domesticus* L.), and lesser mealworm (*Alphitobius diaperinus* P.) [11].

Research and consideration are still underway to get approval for other insect species. For example, there may be the superworm (*Zophobas morio* F.), another species of darkling beetle (Coleoptera: Tenebrionidae) in the same family as the yellow mealworm. The superworm larvae are characterized by up to 49 g/100 g d.w. of protein and up to 42 g/100 g d.w. of fat, consisting of 44–47% of saturated fatty acids (SFAs), 31–32% of monounsaturated fatty acids (MUFAs), and 22–24% of polyunsaturated fatty acids (PUFAs) [13,14,15]. Besides the nutritional value of superworm larvae, it is also noteworthy that they have the ability to utilize polystyrene [14].

The literature about the impact of ultrasound treatment (US) on the quality of dried insects is heavily limited, and, if there is any, it is mainly concerned with species not authorized in the European Union. For instance, ultrasound-assisted extraction has caused an increase in fat extraction yield from house cricket and yellow mealworm [16] and improved the thermostability and antioxidant activity of fat obtained from soybean hawkmoth [17]. Ultrasound-assisted extraction has also improved protein extraction yield from yellow mealworm, cricket adults, and silkworm pupae without changing the amino acid profile [18]. Mishyna et al. [19] have shown that desert locust protein extracts were characterized by improved solubility, coagulability, and foaming stability, while a reduction in solubility and coagulability in honey bee extracts was observed.

The extant study has focused on the impact of ultrasound treatment on the extraction of fat and protein. To the best of our knowledge, no studies have focused on the impact of ultrasound treatment on the chemical composition, mineral composition, allergenicity, or microbiological quality of dried insects. Therefore, the purpose of this work was to assess the impact of blanching and ultrasound treatment on the quality of the dried superworm larvae employing basic chemical composition, mineral composition, FTIR, allergen content, and microbiological quality. Furthermore, fatty acid composition and nutritional indices of extracted fat have been analyzed.

## 2. Results and Discussion

### 2.1. The Effect of Diverse Pretreatments on Chemical Composition of Superworm Larvae

The chemical composition of freeze-dried superworm larvae is shown in Table 1. Regardless of the pretreatments, all dried insects were characterized by a higher fat content than protein content. A significantly lower protein content was determined for samples after ultrasound pretreatment. This may be due to structural changes that can affect the protein content in the sample. As a result of ultrasound treatment, the structure of phospholipids may be damaged, and some nitrogen-containing compounds (e.g., choline and ortho-phosphocholine) may be released and soluble in the treatment medium [20]. Furthermore, ultrasound treatment modifies the protein structure and leads to the unfolding of the protein chain. Broken hydrogen and hydrophobic bonds can lead to increased interactions between protein and water molecules, resulting in a greater dissolution of protein and lower content in the final material [21].

The dried insects subjected to blanching and US treatment at 20 °C were characterized by a significantly higher fat content than the control material. This may be due to structural changes that may provide better solvent penetration and improve extraction yield [22]. The lowest fat content was found for the material after US treatment at 50 °C. This may be due to the increased hydrophobicity of the protein molecules’ surface, resulting in greater fat molecules binding and lower extractability [21].

However, these differences may be due to the different content of this component in the material as it is a complex biological matrix. Further research is needed to clarify possible interactions between components due to new processing methods.

Compared to our results, a higher protein content and a lower fat content in dried superworm larvae have been determined. Zielińska et al. [14] demonstrated about 48.7 g/100 g d.m. of protein and about 31.3 g/100 g d.m. of fat. In the study performed by Kulma et al. [13], freeze-dried superworm larvae were characterized by 47.0–48.1 g/100 g d.m. of protein and 31.3–36.0 g/100 g d.m. of fat. In turn, Adámková et al. [23] demonstrated a lower protein content (39.0 g/100 g d.m) in convective-dried superworm larvae.

### 2.2. The Effect of Diverse Pretreatments on Fatty Acid Composition of Oil Isolated from Superworm Larvae

**Figure 1:**
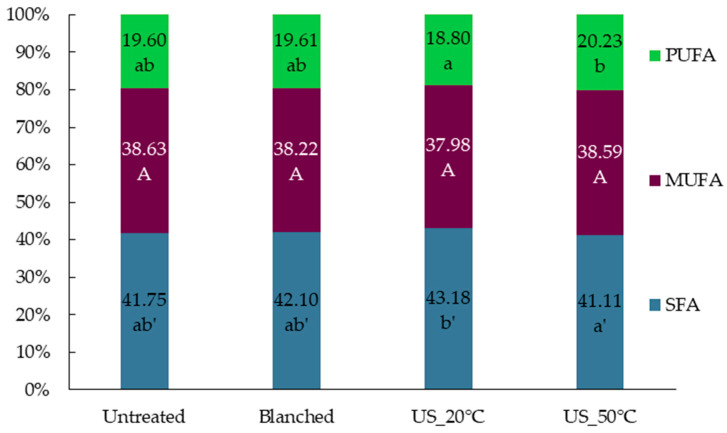
Percentage of fatty acids from the group: saturated fatty acids (SFAs), monounsaturated fatty acids (MUFAs), polyunsaturated fatty acids (PUFAs), and other fatty acids. The different letters (a–b for SFA, A–A for MUFA, and a’–b’ for PUFA) on the bar chart indicate significant difference between samples (Tukey’s HSD test, *p* < 0.05).

The oil from superworm larvae was characterized by a high abundance of saturated SFA (over 41%) and monounsaturated MUFA (over 38%) fatty acids (Figure 1). The US treatment at 20 °C allowed for the lowest polyunsaturated fatty acids content with the highest content of SFA. In turn, increasing the temperature of the US treatment to 50 °C resulted in a significant increase in the content of PUFA.

Fatty acid profiles and calculated health indices of oils extracted from freeze-dried superworm larvae are shown in Table 2. The major fatty acid in the case of all studied oils was oleic acid (35.99–36.87%), followed by palmitic acid (29.74–31.21%). All the oils were also characterized by a high percentage of linoleic acid (17.85–19.15%). Similar fatty acid profiles were previously described by Finke [24], Mattioli et al. [25], and Adamkova et al. [23]. Kulma et al. [13] noted that the fatty acid profile can be dependent on the age of larvae. Based on their results, older larvae (120 days of age) had significantly higher saturated fatty acid content, while MUFA and PUFA percentages were significantly lower compared to 60- and 90-day-old larvae. Compared to the fatty acid profile of other insect larvae, superworm larvae have a higher percentage of SFA than yellow mealworm larvae [23,26,27] but lower than black soldier fly larvae [10,28].

In terms of pretreatment method influence, there were noted some significant differences in the fatty acid percentage share; however, they were not detected in the most abundant fatty acids. The overall observation is that the extraction method can influence the fatty acid composition only to some extent, as the fatty acid profile is a distinctive feature of oil extracted from the same species [22]. Some modifications can be observed, as was detected in the ultrasound-treated samples. Applying ultrasound at a lower temperature (20 °C) resulted in oil with significantly higher PUFA content and lower SFA content than in oil obtained in an ultrasound-assisted procedure at 50 °C. Excessive heating and ultrasound treatment may lead to oil degradation and lipid oxidation [29].

These results correspond with calculated health indices of oils (Table 2), including the atherogenicity index (AI), thrombogenicity index (TI), and the hypocholesterolemic/hypercholesterolemic ratio (HH), all of which are linked to reducing cardiovascular disease risk. Typically, a lower AI (<1.0) and TI (<0.5) and a higher HH (>1.5) suggest a positive health effect, indicating a reduced buildup of atherosclerotic plaque and decreased levels of total cholesterol [30,31]. The values of AI, TI, and HH were the most favorable in the case of oil extracted using sonication at 50 °C. Compared to results obtained by Martins da Silva et al. [32], it can be observed that applying sonication can improve the health indices of oil extracted from superworm larvae. Additionally, the n-6/n-3 fatty acid ratio was calculated. Although the health indices of the superworm larvae oil indicated its high nutritional value, regrettably, the n-6/n-3 fatty acid ratio was higher than recommended (2:1 to 5:1), which may contribute to an increased risk of inflammation-related conditions [30]. Considering the general results of the health indices, superworm larvae could be considered a source of lipids with a higher nutritional value than some fish or dairy products [31].

Based on the gas chromatography results, superworm larvae can be considered as a valuable source of fatty acids and oil with beneficial health indices. The fatty acid profile is partially dependent on the extraction method applied. Alternative extraction methods, like ultrasound-assisted extraction, allow us to obtain oil with improved characteristics. However, the parameters of the extraction procedure have to be carefully selected to achieve the desired effect.

### 2.3. The Effect of Diverse Pretreatments on FTIR Spectra of Superworm Larvae

**Figure 2:**
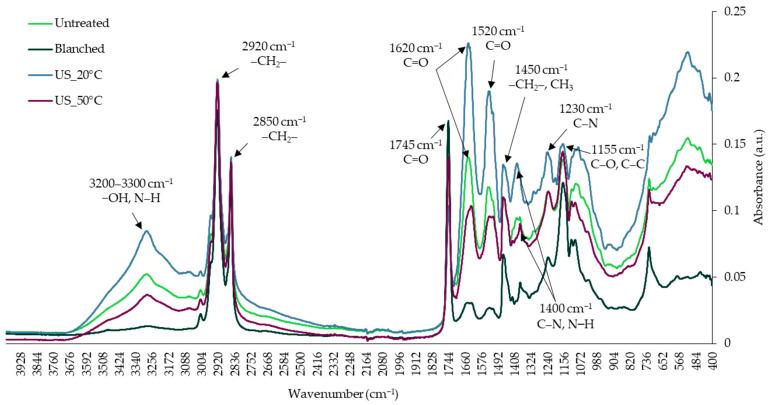
FTIR spectra of freeze-dried superworm larvae after diverse pretreatments.

A similar pattern of obtained FTIR spectra was observed but with a diverse absorbance level (Figure 2). In the region of 3200 to 3300 cm^−1^, a weak stretching vibration is linked to stretching vibrations of the −OH group originating from water molecules and amino acids [33,34] and the N–H bond of the amide A group [35]. A strong peak at 2920 cm^−1^ is related to the asymmetric stretching vibrations of the –CH_2_– group, while a strong peak at 2850 cm^−1^ is responsible for symmetric stretching vibrations of the –CH_2_– group [34]. These strong peaks are linked to the lipids [36] and chitin [34]. Moreover, a peak at 2920 cm^−1^ also indicates the stretching vibration of the C–H bond originating from the amide B group [35]. A strong peak at 1745 cm^−1^ is linked to the stretching vibration of the C=O group of lipids [34,36]. A medium peak at 1620 cm^−1^ is related to the stretching vibration of the C=O group originating from the amide I group [35]. A weak peak at 1520 cm^−1^ indicates amide II, while a medium peak at 1230 cm^−1^ indicates amide III [35,36]. A weak peak around 1450 cm^−1^ is related to the bending vibrations of –CH_2_– and CH_3_ groups, which originate from lipids, proteins [36], and polysaccharides [34]. In turn, a weak peak around 1400 cm^−1^ is related to the stretching vibrations of the C–N bond, and the bending vibrations of the N–H bond originated from amino acids [36]. In the fingerprint region of 1200 to 900 cm^−1^, the stretching vibrations of the C–C, C–O–C, and C–O bonds and the bending vibrations of the C–O–H bond of different carbohydrate groups are visible [35,36]. The region of the FTIR spectrum below the wavenumber of 900 cm^−1^ is associated with conformational changes primarily due to the unique molecular vibrations in the carbohydrates [34,36].

The highest differences between the samples were observed in the range of 3600–3000 cm^−1^ and 1700–1200 cm^−1^ (Figure 2). Higher absorption in these ranges was observed for yellow mealworms dried with the freeze-drying method, which may be related to less damage to the structure and bonds in the molecules than when high temperatures are applied during convective drying. The protein’s secondary structure and, indirectly, its quality correspond mainly to amide I and amide II due to the presence of a sensitive C=O bond [37]. The freeze-dried insects were characterized by a greater absorbance intensity for these peaks than convective-dried insects, which may indicate a better protein secondary structure. However, given the lower protein content (Table 2), it is more probable that the carbonyl groups, which are products of protein and lipid oxidation, are responsible for this phenomenon [38].

### 2.4. The Effect of Diverse Pretreatments on Mineral Composition of Superworm Larvae

Mineral compounds are essential in the human diet, especially those deficiencies such as magnesium, calcium, iron [39], and selenium, which are increasingly seen as deficient elements [40]. In this case, insects also seem to be an interesting source of those compounds. The mineral composition of freeze-dried superworm larvae has shown the presence of those critical minerals (Table 3). The pretreatment used affected the mineral profile of tested insects, and no major tendencies were observed. Generally, US treatment at 20 °C resulted in the greatest changes, and the content of most minerals was significantly lower for this sample. In turn, blanching or US treatment at 50 °C allowed us to obtain comparable or higher contents of individual minerals than untreated insects. Only in the case of magnesium the use of US treatment resulted in a lower content of this macromineral compared to the control sample.

The data on the mineral composition of dried superworm larvae are limited. According to Finke [24], superworm larvae were characterized by 26.2 mg of Ca, 43.5 mg of Mg, 2.0 mg of Fe, and 3.0 mg of Zn. The contents of minerals were lower than those found in our study. Dragojlović et al. [41] found a lower content of Ca (38.7–66.9 mg), Mg (72.0–101.0 mg), and Zn (5.3–7.5 mg) but a higher content of Fe (4.0–11.6 mg). Compared to results obtained in the current study, Mihaly Cozmuta et al. [27] demonstrated a higher amount of Mg (324.4 mg), Ca (225.1 mg), Zn (53.1 mg), and Fe (27.2 mg) in yellow mealworm powder and a lower amount of Mg (51.9 mg) and a higher content of Ca (123.7 mg), Zn (16.6 mg), and Fe (5.8 mg) in house cricket powder in comparison to the current study. Similarly, a higher content of Mg (260.8–305.4 mg), Ca (65.5–73.2 mg), Zn (17.2–20.0 mg), Fe (4.9–6.3 mg), and Se (0.27–0.28 mg) was found in blanched and freeze- or convective-dried yellow mealworm larvae [26]. In general, taking a look at the results obtained and available in the literature, it can be said that the mineral profile depends on many factors, including the species of the insect, diet, age of the insect, processing method, content of other components such as protein or water, etc. [24,41,42,43].

As insects are considered an alternative source of protein and an answer to the need to reduce beef consumption, hence the comparison to this raw material. For instance, Oz et al. [42] provided a higher content of Fe (51.0–58.0 mg), Mg (687.1–836.1 mg), Ca (926.8–1083.3 mg), and Zn (316.8–381.6 mg) for beef steaks cooked with different methods. In turn, Pistón et al. [44] demonstrated a comparable content of Zn (9.5–24.6 mg) and a higher content of Fe (5.6–11.3 mg) in the beef cuts. Nevertheless, insects can be considered a promising alternative iron, zinc, and selenium source in the human diet.

### 2.5. The Effect of Diverse Pretreatments on Allergen Content of Superworm Larvae

Allergens in food are a severe threat to people who are sensitive to them. In the context of insect proteins, there is not yet an adequate regulation of their effects as “primary sensitization”, but it is accepted that pan-allergens are widely distributed in invertebrate groups belonging to a limited number of protein families [45]. The most essential and widely described insect allergen is tropomyosin, which is the most relevant source of it, as an officially approved allergen is presented in crustaceans and mollusks [45,46]. Therefore, taking these facts into account and cross-relationships between arthropods (crustaceans and insects), the examination of the allergenicity of insects using tests for crustacean and mollusk allergens gives a realistic picture of the allergenic behavior of edible insects during processing and makes it possible to determine their potential danger for sensitive individuals [47,48]. Food processing can affect the solubility and immunoreactivity of insect allergens—this is linked to the processing method and the insect species. Protein hydrolysis can sometimes abolish the allergenicity of insects, and various methods—enzymatic, chemical, or physical, such as ultrasound or thermal treatment—can be used to induce this [45,49].

**Figure 3:**
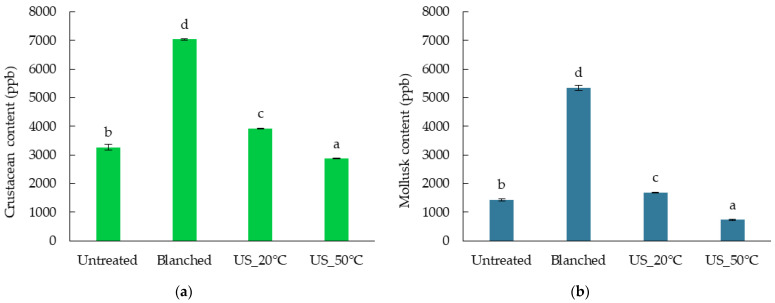
The content of (**a**) crustacean and (**b**) mollusk allergen in freeze-dried superworm larvae after diverse pretreatments. Different letters on the bar chart indicate a significant difference between samples (Tukey’s HSD, *p* < 0.05).

The allergenicity of freeze-dried superworm larvae is presented in Figure 3. Blanched samples exhibited the highest allergen content (7036.23 ppb for crustaceans and 5340.90 ppb for mollusks). Such a short heat treatment may contribute to changes in protein structure, making proteins more accessible. In the case of insects, it may have resulted in a more accessible release and extraction of allergenic proteins from protein aggregates that did not have time to start being hydrolyzed due to the increased temperature and ultrasound treatment [50].

The results show that for both allergens (crustaceans and mollusks), the lowest levels were detected in insects pretreated with the US at 50 °C (2869.09 ppb for crustaceans and 735.29 ppb for mollusks) than in insects pretreated with the US at 20 °C (3924.51 ppb for crustaceans and 1690.24 ppb for mollusks). This is most likely due to the successfully induced hydrolysis of bonds in proteins, which begins to occur more intensively at higher temperatures and additional ultrasonic treatment. Ultrasound can effectively reduce allergen activity by physically destroying proteins, and a combination of several methods may be particularly beneficial [51].

The effect of temperature on the decrease in allergenicity of a food product must be determined for each one and depends on many factors—some allergens are not sensitive even to much higher temperatures than used in this research. This can be seen in the group treated at 20 °C, which fared worse in terms of allergen content than untreated samples (3924.51 ppb vs. 3263.87 for crustaceans and 1690.24 ppb vs. 1424.80 ppb for mollusks), where the temperature was probably too low for protein hydrolysis to begin but already high enough to cause easier extraction.

### 2.6. The Effect of Blanching and Ultrasound Pretreatment on Microbiological Quality of Superworm Larvae

Microbiological stability is one of the criteria for allowing a product to be placed on the market. In the case of edible insects, it depends on the method of rearing and the type of food, as well as on the conditions of transport, and, at the same time, it also depends on the type of insects themselves [52]. Microorganisms can contaminate both the surface of insects, which are easier to remove, and the digestive tract, from which they are impossible to remove. Therefore, innovative methods of processing edible insects are sought, which will reduce the level of microbiological contamination. The superworm larvae were characterized by good microbiological quality. The total count of bacteria (TVC) was at the level of 3.2 log CFU/g, and no Enterobacteriaceae group or anaerobic spore-forming bacteria were observed (Table 4). The contamination with aerobic spore-forming bacteria that could be present on the surface of insects was at the level of 1.5 log CFU/g, while the level of fungi was at 1.1 log CFU/g. Applying the blanching process allowed for reducing TVC by 1 log cycle and applying ultrasound treatment by almost 2 log cycles. These processes did not affect the changes in the numbers of other groups of microorganisms.

The research by Grabowski et al. [52] indicates the possibility of reducing the count of microorganisms in superworm during drying at 80 °C; however, as the authors point out, the microorganisms are often inactivated and not killed, which may lead to their growth in the case of rehydration of the product. Mattioli et al. [25] demonstrated a much higher microbial load of superworm larvae, TVC at 7.2 log CFU/g, and Enterobacteriaceae at 7.5 log CFU/g. This may be related to the method of rearing the insects and their diet, as well as the time of the delivery of the insects to the laboratory for testing. Another study showed that blanching reduced the microbial contamination (TVC) level of superworm larvae by about 2 log cycles [53], regardless of the blanching temperature (60–90 °C). Still, the initial TVC level was much higher than in the present study. The positive effect of blanching on the microbiological quality of other insect species was also demonstrated. In the study conducted by Mancini et al. [54], blanching at 60 °C for 5 min was the most effective in reducing microbiological contamination without negatively affecting the nutrients of yellow mealworms. Similar results were obtained after blanching at 100 °C for 5 min in the study by Ribeiro et al. [55], obtaining a reduction in TVC by 1.7 log cycles and a reduction in Enterobacteriaceae by 5.5 log cycles. These results may indicate that those microorganisms were present on the surface of insects. The effect of ultrasound treatment on the microbiological quality of superworm larvae has not been studied so far.

## 3. Materials and Methods

### 3.1. Material

Live, fasted superworm larvae (*Zophobas morio* F.) were sourced from a local Turkish supplier (Antalya Çekirge, Antalya, Turkey). Experiments were conducted on the larvae on the same day they were acquired. Prior to testing, the larvae were gently rinsed by immersion in tap water and then dried using filter paper.

### 3.2. Technological Treatment

#### 3.2.1. Blanching

Blanching (BL) was carried out by immersing superworm larvae in boiling tap water (98 °C) for 5 min, maintaining a water-to-larvae ratio of 10:1. Following blanching, the larvae were strained through a sieve, cooled in cold tap water for 30 s, and then dried with filter paper. This procedure was performed in duplicate.

#### 3.2.2. Ultrasound Treatment

Ultrasound treatment (US) was conducted using an Elmasonic E100H ultrasonic bath (Elma Schmidbauer GmbH, Singen, Germany) with a 9.5 L capacity (internal tank dimensions: 300 × 240 × 150 mm). The ultrasonic frequency was set to 37 kHz, with an ultrasound power of 100 W and a heating power of 400 W. Treatment duration was 30 min, with initial water temperatures in the ultrasonic bath chamber set to either 20 °C or 50 °C. A tap water-to-larvae ratio of 4:1 was maintained. After treatment, larvae were dried using filter paper. Each ultrasound treatment was performed in duplicate.

#### 3.2.3. Freeze-Drying

Approximately 100 g of both untreated and pretreated superworm larvae were initially frozen at –80 °C for 4 h in a Revco Ultra-Low Freezer ULT1786-5-V39 (Revco, Asheville, NC, USA). The larvae were then freeze-dried for 5 days using a Labconco FreeZone 6 system (model 7753030, Labconco Corp., Kansas City, MO, USA) at a pressure of 0.027 mBar and a condenser temperature of −54 °C. After drying, the samples were packed in a barrier bag.

### 3.3. Chemical Analyses

#### 3.3.1. Basic Chemical Composition

The rapid Soxhlet extraction apparatus Soxtherm SE 416 (Gerhardt, Königswinter, Germany) and n-hexane as a solvent were used to determine fat content, per the manufacturer’s instructions [56]. The protein content was determined using the Kjeldahl method. After mineralization performed using the Kjeldatherm digesting block (Gerhardt, Königswinter, Germany) at 420 °C for 4 h, a fully automated VAPODEST 50s distillation system (Gerhardt, Königswinter, Germany) with an integrated titration unit was used. The protein content was calculated using the nitrogen-to-protein conversion factor 4.76 [56]. The ash content was determined using a muffle furnace Protherm PLF120/5 (Protherm Furnace, Ankara, Turkey) at 550 °C until constant weight was achieved. The moisture content was determined by the oven method using a DHG-9240A oven (Yiheng Inc., Shanghai, China) for 17 h at a temperature of 105 °C until constant weight was achieved [25].

#### 3.3.2. Fatty Acid Composition of Oils

The Folch method was followed to isolate oil from freeze-dried superworm larvae [57]. The fatty acid methyl esters (FAMEs) were derived by transesterification of extracted oil according to the ISO 12966-2:2017 method [58]. FAMEs were analyzed using a Shimadzu GC-2010 gas chromatograph with a flame ionization detector (FID) and fitted with an RT^®^ 2560 silica column (100 m × 0.25 mm inner diameter, 0.20 μm film thickness, RESTEK, Bellefonte, PA, USA). The separation of FAMEs was performed with the following parameters: 140 °C for 5 min, 48 °C/min to 240 °C, and 240 °C for 30 min. The injector and detector temperatures were set at 240 °C and 260 °C, respectively. Helium with a 1.0 mL/min flow rate was used as a carrier gas. The split ratio was 80:1, and the injection volume was 1 μL [59]. FAMEs were identified by comparison of their retention times with the Supelco^TM^ 37 Component FAME mix standard (Sigma-Aldrich GmbH, Schnelldorf, Germany). The percentage content of given fatty acids was established based on the area in the chromatogram. Each insect oil sample was analyzed in triplicate.

#### 3.3.3. Health Indices of Oils

The atherogenicity index (AI), the thrombogenicity index (TI), and the hypocholesterolemic/hypercholesterolemic (HH) ratio were calculated [26,30]:

(1) $$AI=\frac{C12:0+4\times C14:0+C16:0}{\sum MUFA+\sum PUFA}$$

(2) $$TI=\frac{C14:0+C16:0+C18:0}{\frac{\sum MUFA}{2}+\frac{\sum n-6PUFA}{2}+(3\times \sum n-3PUFA)+\frac{n-3PUFA}{n-6PUFA}}$$

(3) $$HH=\frac{C18:1n-9+\sum PUFA}{C12:0+C14:0+C16:0}$$

### 3.4. Mineral Composition

About 0.5 g of the ground-dried insects were placed in digestion tubes, mixed with 10 mL of nitric acid (VWR Chemicals, Darmstadt, Germany) and 2 mL of 30% hydrogen peroxide, and allowed to predigest in an open state for 15 min. The tubes were then sealed, and samples were digested using a MARS6 microwave digestion system (CEM, Matthews, NC, USA) with the following parameters: temperature of 210 °C, microwave power of 1800 W, a 20 min ramp time, and a 15 min hold time. Once cooled to room temperature, samples were filtered through a 0.22 μm syringe filter (Isolab, Laborgeräte GmbH, Eschau, Germany) and diluted with distilled water to obtain aliquots [26]. Mineral concentrations were then measured using an Agilent 7850 ICP-MS system (Agilent Technologies, Santa Clara, CA, USA) with high-purity argon as the plasma gas (15.0 L/min), nebulizer gas (1.08 L/min), and auxiliary gas (0.90 L/min). The results were compared against a certified ICP-MS Calibration Standard (UNSPSC Code 41116107, Agilent Technologies, Santa Clara, CA, USA). Each insect sample was analyzed in triplicate.

### 3.5. FTIR Measurement

A Spectrum Two UATR spectrometer (PerkinElmer, Waltham, MA, USA), equipped with a Universal Attenuated Total Reflectance (UATR) accessory, was used for analysis. A small sample of the ground-dried insects was placed on the crystal and secured with a pressure clamp. Scanning was conducted over a spectral range of 400–4000 cm^−1^ with a resolution of 4 cm^−1^, performing 20 scans per spectrum [26]. The analytical data were recorded and processed using Spectrum 10 software (PerkinElmer, Waltham, MA, USA).

### 3.6. Allergen Content

Approximately 1 g of ground-dried insect material was placed in Falcon tubes, mixed with 20 mL of extraction buffer, incubated for 15 min at 40 °C, then centrifuged at 2000× *g* for 10 min, and filtered through filter paper. Following filtration, 100 μL of the resulting filtrate was added to each well according to the manufacturer’s ELISA protocol (Demeditec Diagnostics GmbH, Kiel, Germany). Absorbance was measured at 450 nm (reference wavelength: 620 nm) using a BioTek™ 800TS microplate reader (BioTek, Winooski, VT, USA). Calibration curves for crustacean and mollusk tropomyosin (0–400 ppb) were utilized [12]. Each insect sample was analyzed in triplicate.

### 3.7. Microorganism Analysis

The ground insects (10 g) were mixed with 90 mL 0.85% NaCl and then homogenized (Stomacher 400 Circulator, Cambridge, UK) for 30 s. Total viable count (TVC) was enumerated on plate count agar (PCA) incubated at 30 °C for 72 h [60]. Yeasts and molds (TYMC) were counted on Dichloran Glicerol DG 18 (DG18) agar after incubation at 25 °C for 120 h [55]. The Enterobacteriaceae group (EG) was enumerated on violet red bile glucose agar (VRBGA) incubated at 37 °C for 24 h [55]. Bacterial endospores were enumerated by heat shocking insect dilution for 20 min at 80 °C in a sterile tube, after which pour plates with agar counts (PCA for aerobic or Wilson Blair agar for anaerobic spore-forming bacteria) were incubated at 30 °C for 48 h [12]. The number of microorganisms was counted using ProtoCOL 3-device (Synbiosis, Frederick, MD, USA) and determined in log CFU/g. All determinations were conducted in triplicates. All media were purchased from Biomaxima, Poland.

### 3.8. Statistical Analysis

The one-way ANOVA procedure (α = 0.05) and the post hoc Tukey’s HSD test were followed using STATISTICA 13.3 (TIBCO Software, Palo Alto, CA, USA) to assess the significant differences between the investigated properties.

## 4. Conclusions

The study proved that blanching (BL) and ultrasound (US) as pretreatments used before the freeze-drying process influenced the chemical and microbiological characteristics of the freeze-dried superworm larvae that were obtained.

The blanching allowed the highest protein content in the dried insects to be obtained, while a significantly lower protein content in insects after the US application was observed compared to the untreated insects. Using blanching and US treatment at 20 °C resulted in a significantly higher fat content in the dried insects compared to other samples.

Oils isolated from freeze-dried superworm larvae comprised comparable amounts of saturated (over 41%) and monounsaturated (over 38%) fatty acids. Linoleic (C16:0) and oleic (C18:1 n-9c) fatty acids dominated the fatty acid composition of each insect oil. Based on the fatty acid profile and calculated health indices, oil extracted from superworm larvae subjected to US treatment at 50 °C exhibited the most beneficial health-promoting properties.

The use of blanching did not result in significant changes in mineral content. Insects subjected to US treatment at 20 °C (US_20°C) exhibited the lowest content of most minerals tested. Only the selenium content was significantly higher in the US-treated insects than in the untreated and blanched insects.

The blanching allowed the highest crustacean and mollusk contents in the dried insects to be obtained. Only the US application at 50 °C provided a lower crustacean content and mollusk allergen content compared to the untreated insects.

The dried superworm larvae exhibited good microbiological quality, e.g., the absence of the Enterobacteriaceae group and anaerobic spore-forming bacteria. Using both pretreatment methods allowed for reducing the total viable count. Blanching has a lower effect in reducing TVC (by 1 log cycle) than ultrasound treatment (by almost 2 log cycles).

Further research should focus on the protein nutritional value (amino acid profile and digestibility) and functional properties such as water and oil-holding capacity, solubility, and emulsification. Furthermore, the antioxidant and anti-inflammatory activities of peptide fractions should be considered.

## Figures and Tables

**Table 1:** Chemical composition of freeze-dried superworm larvae after diverse pretreatments.

Component (g/100 g d.m.)	Untreated	Blanched	US_20°C	US_50°C
Moisture	3.68 ± 0.12 a ^1^	4.07 ± 0.01 b	3.78 ± 0.08 ab	3.84 ± 0.11 ab
Protein	36.38 ± 1.28 b	37.72 ± 1.56 b	31.65 ± 0.96 a	33.34 ± 0.13 a
Fat	41.29 ± 0.77 a	42.84 ± 1.13 b	42.80 ± 0.27 b	40.95 ± 0.09 a
Ash	2.56 ± 0.09 a	2.38 ± 0.06 a	2.47 ± 0.07 a	2.43 ± 0.02 a

^1^ Different letters in the row indicate a significant difference between samples (Tukey’s HSD test, *p* < 0.05).

**Table 2:** Fatty acid composition and health indices of oil extracted from freeze-dried superworm larvae after diverse pretreatments.

Fatty Acid (%)	Untreated	Blanched	US_20°C	US_50°C
Caprylic acid (C8:0)	0.22 ± 0.01 a ^1^	0.47 ± 0.03 c	0.24 ± 0.01 a	0.32 ± 0.03 b
Capric acid (C10:0)	0.12 ± 0.01 a	0.15 ± 0.01 b	0.12 ± 0.01 b	0.12 ± 0.01 a
Lauric acid (C12:0)	0.04 ± 0.00 a	0.07 ± 0.01 b	0.03 ± 0.00 a	0.04 ± 0.00 a
Myristic acid (C14:0)	0.86 ± 0.04 b	1.23 ± 0.03 c	0.89 ± 0.02 b	0.77 ± 0.03 a
Pentadecanoic acid (C15:0)	0.26 ± 0.01 a	0.30 ± 0.01 b	0.28 ± 0.02 ab	0.25 ± 0.01 a
cis-10-Pentadecenoic acid (C15:1)	0.02 ± 0.00 b	0.02 ± 0.00 b	0.02 ± 0.00 b	0.01 ± 0.00 a
Palmitic acid (C16:0)	30.54 ± 0.64 a	31.21 ± 0.77 a	30.95 ± 0.58 a	29.74 ± 0.56 a
Palmitoleic acid (C16:1)	1.43 ± 0.04 b	1.61 ± 0.05 c	1.56 ± 0.04 c	1.28 ± 0.02 a
Heptadecanoic acid (C17:0)	0.92 ± 0.03 ab	0.96 ± 0.02 b	0.95 ± 0.03 b	0.87 ± 0.02 a
cis-10-Heptadecenoic acid (C17:1)	0.26 ± 0.01 b	0.25 ± 0.01 ab	0.23 ± 0.01 a	0.23 ± 0.01 a
Stearic acid (C18:0)	8.08 ± 0.15 b	7.32 ± 0.19 a	8.95 ± 0.21 c	8.31 ± 0.18 b
Elaidic acid (C18:1 n-9t)	0.04 ± 0.00 a	0.06 ± 0.00 c	0.04 ± 0.00 a	0.05 ± 0.00 b
Oleic acid (C18:1 n-9c)	36.74 ± 0.53 a	36.15 ± 0.77 a	35.99 ± 0.41 a	36.87 ± 0.59 a
Linoleic acid (C18:2 n-6c)	18.53 ± 0.21 ab	18.58 ± 0.33 ab	17.85 ± 0.49 a	19.15 ± 0.28 b
γ-Linolenic acid (C18:3 n-6)	0.04 ± 0.00 b	0.05 ± 0.00 c	0.03 ± 0.00 a	0.04 ± 0.00 b
α-Linolenic acid (C18:3 n-3)	0.71 ± 0.01 b	0.80 ± 0.02 d	0.65 ± 0.00 a	0.76 ± 0.02 c
Arachidic acid (C20:0)	0.22 ± 0.00 b	0.17 ± 0.00 a	0.29 ± 0.01 c	0.18 ± 0.00 a
cis-11-Eicosenoic acid (C20:1 n-9c)	0.13 ± 0.00 c	0.11 ± 0.00 a	0.12 ± 0.00 b	0.13 ± 0.00 c
cis-11,14-Eicosadienoic acid (C20:2) + Henicosanoic acid (C21:0)	0.06 ± 0.00 c	0.04 ± 0.00 a	0.04 ± 0.00 a	0.05 ± 0.00 b
cis-8,11,14-Eicosatrienoic acid (C20:3 n-6)	0.20 ± 0.01 c	0.10 ± 0.01 a	0.18 ± 0.02 bc	0.16 ± 0.01 b
Arachidonic acid (C20:4 n-6) + cis-11,14,17-Eicosatrienoic acid (C20:3 n-3)	0.02 ± 0.00 b	0.01 ± 0.00 a	0.01 ± 0.00 a	0.03 ± 0.00 c
Behenic acid (C22:0)	0.23 ± 0.01 b	0.15 ± 0.00 a	0.27 ± 0.02 c	0.26 ± 0.01 bc
cis-5,8,11,14,17 Eicosapentaenoic (C20:5 n-3)	0.01 ± 0.00 a	0.01 ± 0.00 a	0.02 ± 0.00 b	0.01 ± 0.00 a
cis-13,16-Docosadienoic acid (C22:2)	0.05 ± 0.00 a	0.05 ± 0.00 a	0.06 ± 0.00 b	0.07 ± 0.00 c
Lignoceric acid (C24:0)	0.26 ± 0.01 c	0.07 ± 0.00 a	0.21 ± 0.02 b	0.25 ± 0.00 c
Nervonic acid (C24:1)	0.01 ± 0.00 a	0.02 ± 0.00 b	0.02 ± 0.00 b	0.02 ± 0.00 b
cis-4,7,10,13,16,19 Docosahexaenoic acid (C22:6 n-3)	0.03 ± 0.00 b	0.02 ± 0.00 a	0.02 ± 0.00 a	0.03 ± 0.00 b
	Health Indices			
n-6/n-3	25.74 ± 0.42 bc	23.15 ± 0.97 a	27.38 ± 0.71 c	24.54 ± 0.56 ab
Atherogenicity index (AI)	0.58 ± 0.01 ab	0.63 ± 0.01 c	0.61 ± 0.02 bc	0.56 ± 0.00 a
Thrombogenicity index (TI)	1.28 ± 0.02 a	1.28 ± 0.02 a	1.36 ± 0.04 b	1.24 ± 0.01 a
Hypocholesterolemic/hypercholesterolemic ratio (HH)	1.79 ± 0.04 ab	1.72 ± 0.03 a	1.72 ± 0.06 a	1.87 ± 0.02 b

^1^ Different letters in the row indicate significant differences between samples (Tukey’s HSD, *p* < 0.05).

**Table 3:** Mineral composition of freeze-dried superworm larvae after diverse pretreatments.

Mineral (mg/ 100 g d.m.)	Untreated	Blanched	US_20°C	US_50°C
Potassium (K)	1056.24 ± 11.32 b ^1^	1065.12 ± 17.17 b	1006.36 ± 8.16 a	1050.90 ± 15.36 b
Sodium (Na)	127.48 ± 1.26 b	126.46 ± 2.47 b	126.29 ± 1.86 b	118.09 ± 1.65 a
Magnesium (Mg)	122.84 ± 2.90 b	124.55 ± 1.87 b	111.94 ± 1.73 a	114.07 ± 2.04 a
Calcium (Ca)	64.33 ± 9.89 a	78.80 ± 5.00 a	65.45 ± 0.85 a	76.23 ± 6.14 a
Zinc (Zn)	12.69 ± 0.30 a	13.92 ± 0.34 b	12.70 ± 0.13 a	13.48 ± 0.34 b
Iron (Fe)	4.80 ± 0.11 c	4.47 ± 0.14 b	3.93 ± 0.13 a	4.37 ± 0.05 b
Copper (Cu)	1.37 ± 0.04 b	1.42 ± 0.03 b	1.20 ± 0.03 a	1.41 ± 0.03 b
Manganese (Mn)	1.02 ± 0.03 b	1.16 ± 0.03 c	0.82 ± 0.02 a	1.09 ± 0.03 b
Selenium (Se)	0.14 ± 0.01 a	0.15 ± 0.01 b	0.17 ± 0.00 c	0.21 ± 0.01 d

^1^ Different letters in the row indicate significant differences between samples (Tukey’s HSD, *p* < 0.05).

**Table 4:** Microbiological quality of superworm larvae after diverse pretreatments.

Microorganism (log CFU/g)	Untreated	Blanched	US_20°C	US_50°C
Total viable count (TVC)	3.24 ± 0.16	2.25 ± 0.03	1.56 ± 0.34	1.48 ± 0.17
Enterobacteriaceae group (EG)	≤1.00	≤1.00	≤1.00	≤1.00
Aerobic spore-forming bacteria	1.56 ± 0.05	1.49 ± 0.02	1.59 ± 0.10	1.31 ± 0.08
Anaerobic spore-forming bacteria	≤1.00	≤1.00	≤1.00	≤1.00
Total yeast and mould count (TYMC)	1.10 ± 0.12	1.21 ± 0.07	1.35 ± 0.18	1.05 ± 0.03

## Data Availability

The original contributions presented in the study are included in the article. Further inquiries can be directed to the corresponding authors.
